# ViMG: a contactless deep learning framework for estimating sEMG amplitude envelopes from facial videos

**DOI:** 10.3389/fbioe.2026.1945313

**Published:** 2026-10-30

**Authors:** Faraz A. Nizamani, Mayur J. Bhamborae, Sophie Bonnaire, Farah I. Corona-Strauss, Daniel J. Strauss

**Affiliations:** 1 Systems Neuroscience and Neurotechnology Unit, Faculty of Medicine, Saarland University & School of Engineering, htw saar, Homburg/Saar, Germany; 2 Center for Digital Neurotechnologies Saar (CDNS), Saarbrücken, Germany

**Keywords:** affective computing, contactless sensing, deep learning, facial expression analysis, surface electromyography (sEMG), video myography

## Abstract

Traditional surface electromyography relies on contact electrodes that can restrict natural facial movements and cause discomfort. These limitations are particularly critical in cognitive and developmental research, where sensor placement can alter the very behavior being observed, especially in longitudinal monitoring. We present Video Myography (ViMG), an approach for the contactless estimation of window-averaged zygomaticus major sEMG envelope amplitude from facial video data. Rather than reconstructing the raw sEMG waveform, ViMG predicts a single scalar per 0.7-s video window, corresponding to the mean amplitude of the processed root-mean-square sEMG envelope over that window. Our method employs a hybrid 3D U-Net architecture integrated with a Long Short-Term Memory network intended to capture spatiotemporal dynamics of perioral facial movements. On a dataset of 23 subjects recorded in a controlled laboratory setting, we estimate window-averaged sEMG envelope amplitude over short temporal windows, achieving a mean Pearson correlation coefficient of 0.661 (range across the five folds: 0.536–0.872). A landmark-based baseline achieved comparable performance (r=0.670) , indicating that the recoverable signal is largely explained by coarse perioral kinematics and can be captured by substantially simpler models. Gradient-based saliency maps indicate that the model’s predictions are informed by anatomically plausible perioral regions. While currently limited to controlled environments, this work establishes a proof-of-concept for contactless physiological assessment and a starting point for future contactless monitoring systems.

## 1 Introduction

Facial surface electromyography (sEMG) is widely used in numerous fields (Merletti and Farina, 2016), from medical diagnostics and rehabilitation (Barry et al., 1990; Vigotsky et al., 2018) to human-computer interaction (Qi et al., 2019) and emotion recognition (Hamedi et al., 2011). By measuring the electrical activity of facial muscles (Hermens et al., 2000), particularly the zygomaticus major muscle responsible for smile formation (Iwanaga et al., 2022; Zabojova et al., 2018), sEMG provides valuable insights into facial expressions (Jiang et al., 2015), emotional states (Van Boxtel, 2010), and neuromuscular disorders (Hogrel, 2005; Rau and Disselhorst-Klug, 1997). However, the conventional approach of using contact-based electrodes presents significant limitations (Chi et al., 2010) that can compromise the natural dynamics of facial expressions (Inzelberg et al., 2018a) and the quality of long-term monitoring data (Inzelberg et al., 2018b). The presence of physical electrodes on the face can interfere with natural facial movements (Ibrahim et al., 2024) and may cause discomfort during prolonged use (Inzelberg et al., 2018b). This limitation is particularly problematic in studies of spontaneous facial expressions or emotions (Hietanen et al., 2019), where awareness of sensor placement might influence the authenticity of expressions, as well as in cognitive and developmental systems research. In developmental studies involving children or individuals with neurodevelopmental conditions (e.g., Autism Spectrum Disorder), the application of tactile sensors can induce sensory distress, leading to behavioral artifacts (Marco et al., 2011) that obscure the cognitive states under investigation. These difficulties compound in pediatric populations, where the reduced scale of facial anatomy shortens inter-electrode and inter-muscle distances, making standardized electrode placement harder to achieve and increasing the likelihood that a given electrode pair registers volume-conducted activity from adjacent mimetic muscles. In cognitive load monitoring, the awareness of attached sensors can act as a confounding variable. Therefore, a truly contactless method is required. While current vision-based solutions are still sensitive to environmental factors, establishing a baseline correlation in controlled settings is the necessary first step toward this goal.

For contactless prediction of zygomaticus major activity in particular, the primary difficulties are the inherently low signal-to-noise ratio of camera-based measurements and the sensitivity to motion artifacts, which are difficult to avoid without algorithmic correction, as subjects must remain perfectly still (McDuff, 2021). These challenges are particularly acute due to the difficulty in isolating the target muscle’s activity from crosstalk from adjacent mimetic muscles, such as the levator labii superioris or risorius (Zhang et al., 2023). This is especially true when dealing with mean sEMG envelope amplitude during spontaneous and subtle smiles, which produce minute signal changes that are difficult to distinguish from both system noise and the activity of neighboring muscles, even under controlled laboratory illumination (Tassinary and Cacioppo, 1992; Fridlund and Cacioppo, 1986).

In this paper, we introduce ViMG (Video Myography), a deep learning framework designed to estimate the window-averaged amplitude of the facial surface electromyography (sEMG) envelope from video. The model regresses a single scalar per short video window, corresponding to the mean of the processed RMS sEMG envelope of the zygomaticus major over that window, rather than reconstructing the raw sEMG waveform. While translating visual kinematics into physiological signals has been demonstrated for the large skeletal muscles of the body, it has not been shown for facial musculature; our goal is to establish a proof-of-concept for this translation in the facial domain within a controlled setting. ViMG integrates a 3D U-Net (Çiçek et al., 2016) with a Long Short-Term Memory (LSTM) (Hochreiter and Schmidhuber, 1997) network to capture both spatial and temporal dynamics of facial movements. Our hybrid model uses the 3D U-Net’s encoder-decoder structure to preserve spatial features while the LSTM was intended to model temporal dependencies in facial muscle activation patterns, enabling the model to learn complex spatiotemporal relationships between facial kinematics and underlying muscle activity. By using the spatial capabilities of the 3D U-Net, our model aims to learn deformation patterns associated with zygomaticus major activity. Because the approach is optical rather than electrical, it is not affected by the volume conduction that gives rise to electrical crosstalk in traditional sEMG, although mechanical coupling between adjacent facial muscles remains a source of signal mixing. We also provide an interpretability pipeline using gradient-based saliency maps to spatially resolve sEMG signal contributions, identifying which perioral regions most significantly influence predicted zygomaticus major activity and offering insights into the spatial distribution of muscle activation during mouth movements.

Main Contributions: This work contributes to contactless physiological monitoring by introducing a 3D U-Net-LSTM architecture for camera-based estimation of window-averaged sEMG envelope amplitude, validating the approach on 23 subjects, and providing interpretability analysis that reveals how perioral facial movements relate to zygomaticus major muscle activation.

Section 2 reviews the relevant literature, and Section 3 details our proposed methodology. In Section 4, we present and discuss the experimental results. Section 5 addresses the limitations of our study, Section 6 discusses future directions, and Section 7 provides our conclusions.

## 2 Related work

Facial expression analysis has advanced considerably along two primary pathways: the application of deep learning to computer vision-based approaches (Pereira et al., 2024; Ahmad et al., 2023; Haq et al., 2024; Huang et al., 2023; Kopalidis et al., 2024) and the improvements in surface EMG measurement technologies (Rutkowska et al., 2024; Funk et al., 2024; Franz et al., 2024; Guntinas-Lichius et al., 2023; Kołodziej et al., 2024; Li et al., 2025). However, these computer vision-based methods rely on surface-level visual features for classifying discrete emotions or facial action units from visual data, rather than quantifying the underlying neuromuscular activity that drives the expressions. Conversely, surface electromyography (sEMG) provides a direct measurement of muscle electrical activity (Trentzsch et al., 2020); however, it still requires physical electrode placement that can interfere with natural facial movements and limit long-term monitoring applications (Inzelberg et al., 2018b). The limitations of both approaches-the lack of physiological grounding in computer vision methods and the invasive nature of traditional sEMG systems—have motivated researchers to explore novel contactless approaches that combine the advantages of both modalities while eliminating the need for physical contact with the sensors (Perusquia-Hernandez et al., 2020).

As direct contactless sEMG estimation from video is an emerging area, standardized benchmarks for direct comparison are limited. We therefore ground our contributions by reviewing foundational work in computer vision, electromyography, and relevant deep learning architectures.

### 2.1 Computer vision-based facial expression recognition

Computer vision methods for facial expression recognition have been applied extensively through the application of convolutional neural networks (CNNs) and advanced deep learning architectures. A prominent approach for capturing spatiotemporal dynamics in facial expressions involves hybrid architectures that combine spatial feature extraction with temporal modeling. For instance (Yu et al., 2018), proposed a spatio-temporal Convolutional features with Nested LSTM (STC-NLSTM) architecture that integrates three deep learning sub-networks: a 3D CNN for spatiotemporal feature extraction, a temporal T-LSTM that preserves temporal dynamics, and a convolutional C-LSTM to model multi-level features (Yu et al., 2018).

Within the framework, the 3D CNN extracts spatio-temporal convolutional features from image sequences that represent facial expressions, while expression dynamics are captured through a Nested LSTM architecture comprising two specialized components: a Temporal LSTM (T-LSTM) that models temporal dependencies within spatio-temporal features at each convolutional layer, and a Convolutional LSTM (C-LSTM) that aggregates outputs from all T-LSTMs to encode hierarchical features across network layers. Yu et al. (2018) reported improved performance over existing methods through experiments on four benchmark databases: CK+, Oulu-CASIA, MMI, and BP4D (Yu et al., 2018). This methodology illustrates how 3D convolutional layers can effectively be integrated with recurrent architectures to extract both spatial patterns of facial expressions and track their changes over time, establishing a foundation for spatiotemporal modeling approaches that influenced subsequent research in video-based facial analysis.

Nevertheless, vision-based methods encounter substantial challenges when deployed in real-world scenarios. As highlighted by Cai et al., these methods suffer from “flaws in terms of high requirement of shooting environment” where “orientation of the camera, resolution of image, and lighting condition have a significant influence on the quality of experiment images” (Cai et al., 2018). Additionally, vision-based systems struggle with subtle micro-expressions that produce minimal visible facial changes and exhibit reduced accuracy when facial features are partially occluded or when dealing with non-prototypical facial anatomy. Furthermore, privacy concerns regarding continuous facial monitoring limit its deployment in sensitive environments. These limitations have driven researchers to investigate bio-signal analysis as an alternative or supplementary method to conventional visual approaches.

### 2.2 Electromyography-based facial expression analysis

Facial muscle activity can be effectively analyzed using surface electromyography (sEMG), a technique that can detect small muscle contractions that standard imaging methods might miss. The conventional sEMG process requires placing electrodes directly onto facial muscles to record electrical signals as expressions develop. While this approach delivers accurate, measurable data about how muscles activate, it comes with practical challenges: the electrodes can be difficult to position correctly, may interfere with normal facial movement, and can cause discomfort when used for longer periods of monitoring (Franz et al., 2024).

Recent advances in sEMG technology have focused on developing less intrusive measurement techniques while maintaining signal quality. Wireless high-resolution sEMG systems using smart skin technology have demonstrated the ability to discriminate between different facial expressions with high precision, achieving 82% differentiation accuracy across multiple facial movement tasks (Funk et al., 2024). However, these approaches still require direct skin contact and electrode placement, which can affect the authenticity of natural expressions and limit long-term monitoring applications.

### 2.3 Multi-modal and hybrid approaches

The limitations of individual modalities have led to the development of multimodal approaches that combine computer vision and sEMG techniques to use the strengths of both methods. Perusquia-Hernandez et al. (2020) presented an approach that estimates specific Facial Action Units (AUs) from distal facial sEMG combined with computer vision techniques. Their method uses Independent Component Analysis (ICA) and Non-Negative Matrix Factorization (NNMF) to determine correspondence between muscle synergies and visually observable facial movements. The method achieved 81% accuracy for AU06 (Orbicularis Oculi) and 82% for AU12 (zygomaticus Major), highlighting the effectiveness of integrating sEMG and visual approaches for analyzing facial expressions.

By placing electrodes at a distance from primary facial muscles, this method can record significant muscle signals without being disrupted by spontaneous facial movements. Nevertheless, remote sEMG measurement presents the difficulty of uncertain muscle origin identification, necessitating advanced signal processing and machine learning methods to derive useful insights.

More recently, Shu et al. (2025) recorded synchronized facial video and sEMG from seven facial muscle regions, including the zygomaticus major, and extracted muscle synergies via non-negative matrix factorization to recognize facial expressions, while a skin-musculoskeletal model estimated the displacement of facial keypoints such as the mouth corners and nasolabial folds. Their work reinforces the tight mechanical coupling between zygomaticus major activity and perioral kinematics that ViMG seeks to exploit; however, it operates in the opposite direction to ours, mapping measured sEMG forward onto facial kinematics through a biomechanical model and still requires worn electrodes.

### 2.4 Deep learning architectures for spatiotemporal analysis

The application of deep learning to facial expression analysis has advanced considerably, particularly in developing architectures capable of capturing both spatial and temporal dynamics. Three-dimensional CNNs have proven effective for simultaneous spatial and temporal feature extraction, addressing the limitations of traditional 2D CNNs that can only capture spatial features. The integration of 3D CNNs with recurrent neural networks, particularly LSTM architectures, is now a common approach for modeling complex spatiotemporal relationships in facial expression data.

Recent work has demonstrated that hybrid architectures combining 3D convolutional operations with LSTM networks can effectively model the temporal evolution of facial expressions while maintaining spatial feature representation capabilities. These approaches have shown superior performance compared to methods that treat spatial and temporal modeling as separate sequential processes, highlighting the importance of joint spatiotemporal feature learning in facial expression analysis.

### 2.5 Vision-based estimation of muscle activity

A directly related line of work estimates muscle activity from video. Most relevant is the Muscles in Action (MIA) framework of Chiquier and Vondrick (2023), which introduced a dataset of 12.5 h of synchronized single-view video and sEMG from ten subjects performing whole-body exercises, and learned a mapping between visible motion and muscle activation. MIA regresses continuous sEMG for eight muscle sites and conversely reconstructs body pose from muscle activation, evaluating on both in-distribution and out-of-distribution subjects and exercises. Methodologically, MIA operates on 2D and 3D body keypoints recovered by a pose-estimation model rather than on raw image data, exploiting the well-defined skeletal kinematics of limb movement.

ViMG differs from MIA in domain, input representation, and application. First, we target facial musculature, specifically the zygomaticus major, rather than large limb and torso muscles. Facial sEMG estimation is substantially harder, as the driving cues are subtle perioral skin deformations rather than gross joint displacements, with lower signal amplitude, lower signal-to-noise ratio, and crosstalk from adjacent mimetic muscles. Second, the face lacks the articulated skeleton that pose estimators recover for the body, so the specific skeletal keypoint representation MIA relies on does not transfer directly. Facial landmarks can nonetheless describe perioral displacement, velocity, and deformation, and constitute a plausible alternative representation. We chose instead to learn directly from raw perioral pixel sequences through a 3D U-Net, in order to preserve fine-grained cues such as skin texture change and local bulging that sparse landmark sets do not represent explicitly. Third, our application context is affective and developmental psychophysiology, where removing contact electrodes is motivated by ecological validity and by populations (e.g., infants or individuals with neurodevelopmental conditions) for whom sensor placement is itself a confound. We thus position ViMG not as the first demonstration that muscle activity can be regressed from video, a paradigm established for body musculature by MIA, but as an extension of that paradigm to the facial domain, with an interpretability analysis linking predicted activation to anatomically plausible perioral regions.

### 2.6 Research gap and contribution

Despite significant advances in both computer vision and sEMG-based approaches, a critical gap remains in developing truly contactless methods for estimating muscle activity envelopes that eliminate the need for physical electrode placement while retaining a useful correspondence with the sEMG signal. While recent vision-based methods have demonstrated that muscle activity can be regressed from video, this has been established for the large skeletal muscles of the body rather than for facial musculature. Existing multimodal facial approaches, meanwhile, still require some form of sEMG sensor contact, whether through distal wearable electrodes combined with computer vision (Perusquia-Hernandez et al., 2020) or through electrode-based biomechanical modeling of facial kinematics (Shu et al., 2025), limiting their applicability in scenarios where completely non-contact monitoring is essential. In contrast to Shu et al. (2025), who estimate facial kinematics from measured sEMG, ViMG solves the inverse, fully contactless problem of predicting the sEMG envelope directly from video, with no electrodes required at inference.

Our work addresses this gap by proposing a contactless approach for estimating facial sEMG amplitude envelopes directly from video data using a hybrid 3D U-Net and LSTM architecture. Unlike previous multimodal approaches that combine existing sEMG measurements with computer vision, our method aims to eliminate the need for physical sEMG sensors entirely while estimating a window-averaged sEMG-derived amplitude measure that would typically require direct electrode placement. This represents a step toward contactless physiological monitoring that combines information related to the sEMG envelope with the non-intrusive nature of computer vision approaches.

We adapt a 3D U-Net–LSTM architecture to facial sEMG envelope regression and evaluate it against a learned landmark-based baseline. Our results demonstrate the viability of contactless estimation of facial sEMG envelopes, while also showing that comparable performance is attainable with a substantially simpler model, a finding we report in full in Section 4.4.

We employ gradient-based saliency mapping to provide interpretability for our model’s predictions, revealing which specific regions within the perioral area most strongly influence the predicted zygomaticus major muscle activity. This spatial attention analysis suggests that our model focuses on anatomically relevant regions around the mouth corners and nasolabial folds.

## 3 Methodology

To map surface kinematics to underlying neuromuscular activation, we developed a pipeline that synchronizes high-frame-rate video recordings of facial movements with concurrent sEMG amplitude envelopes derived from the zygomaticus major muscle. Data acquisition was conducted in a single continuous session per subject to minimize electrode impedance variability and ensure temporal consistency between modalities. Our methodology for contactless estimation of window-averaged facial sEMG envelope amplitude comprises four stages: data collection, data processing, model architecture, and model training (Figure 1). The approach applies a hybrid deep learning architecture combining 3D U-Net spatial feature extraction with LSTM temporal modeling to learn the spatiotemporal relationships between observable facial kinematics and underlying muscle activity. Subject-independent cross-validation across all participants was used to assess whether the learned mapping transfers to unseen subjects within this controlled setting.

**FIGURE 1:**
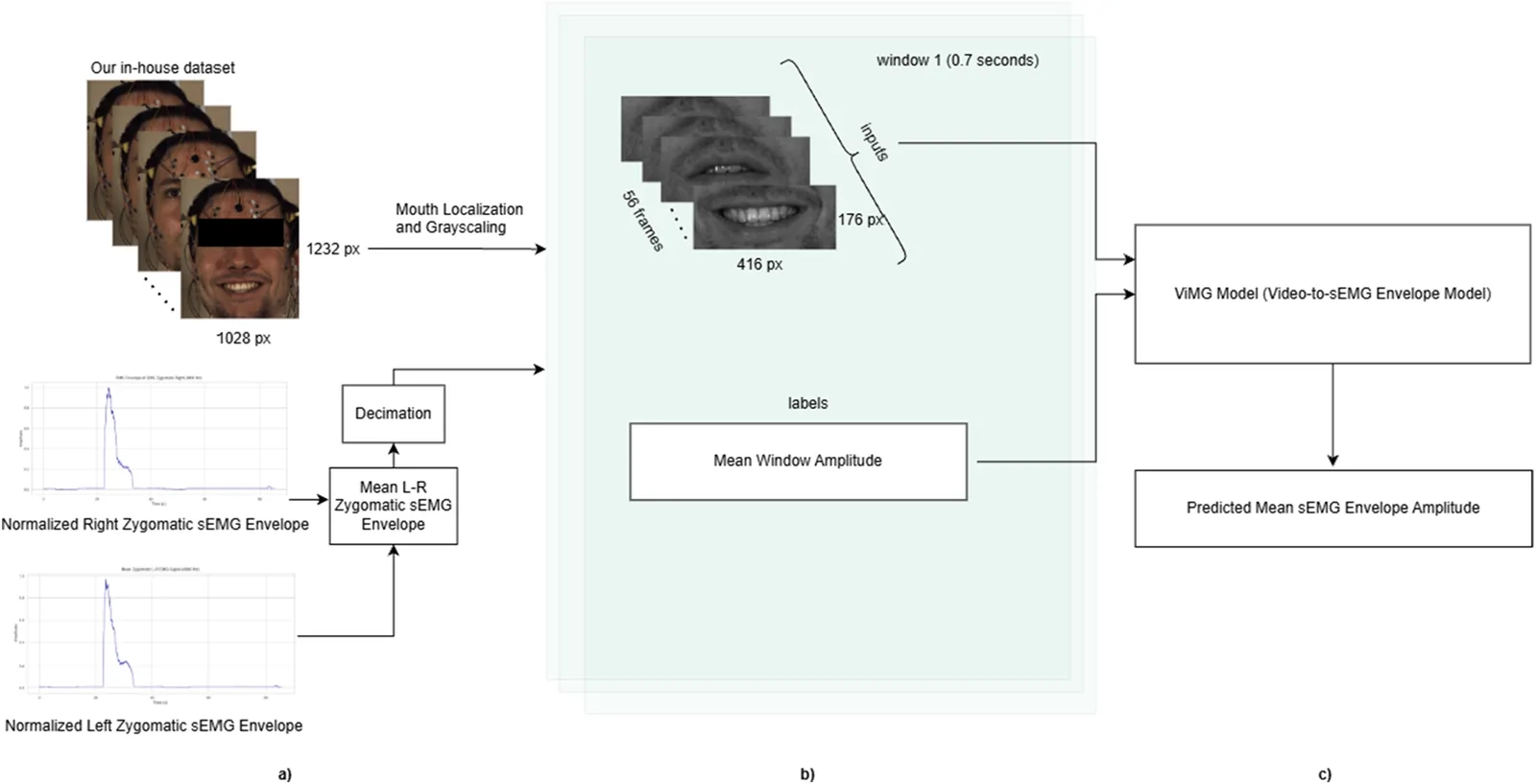
Three-stage pipeline for contactless sEMG envelope prediction. **(a)** Data pre-processing: perioral region extraction from facial videos and sEMG signal normalization and decimation. **(b)** Temporal windowing: synchronization of 56-frame video sequences with the corresponding ground-truth mean sEMG envelope amplitude. **(c)** ViMG model: hybrid 3D U-Net and LSTM architecture that estimates the aggregate muscle activity intensity for each 0.7-s segment.

### 3.1 Data collection

Data were collected in a controlled laboratory setting (Figure 2), where participants, secured with a head fixation device, were monitored by a camera, an eye-tracker, and various physiological sensors while being presented with audiovisual stimuli.

**FIGURE 2:**
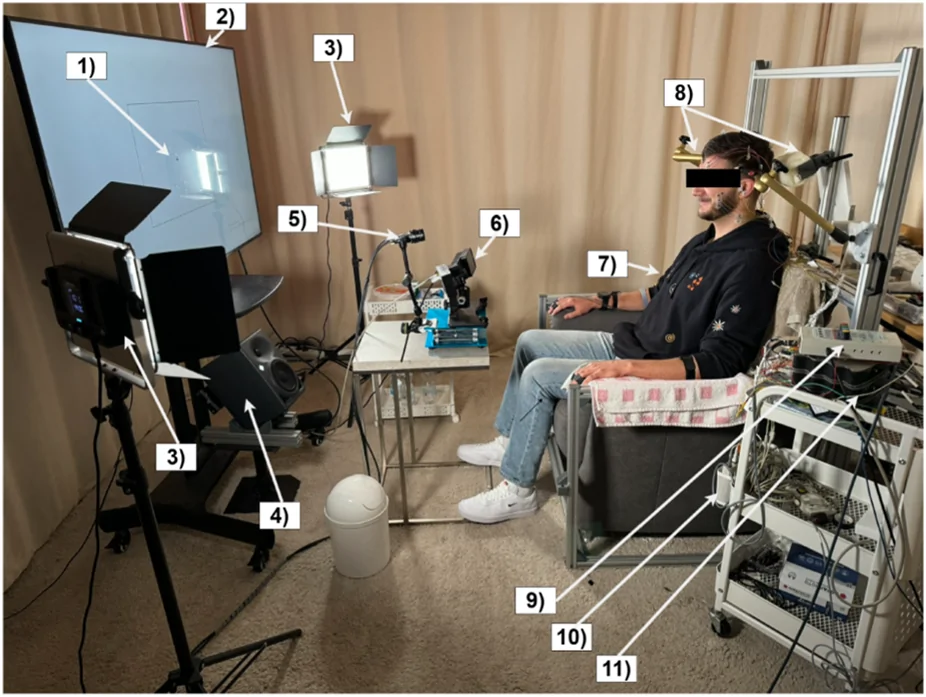
Picture of experimental setup (hardware). Labels: 1) Fixation Cross, 2) TV, 3) Spotlights, 4) Speaker, 5) Camera, 6) Eye tracker, 7) Participant, 8) Head fixation, 9) Trigger box, 10) Signal amplifiers with electrodes and sensors connected, 11) Sensors: EDA, SpO2, and pulse. Components 6 and 11 are part of the shared laboratory setup but were not used in this experiment. Not shown are computers, associated monitors, a function generator, and a sound card.

#### 3.1.1 Participants and setup

From an initial set of 30 subjects (mean age 26.2 ± 3.3 years; 17 males, 13 females), 23 subjects were included in the final analysis. Seven subjects were excluded prior to any model training. The reasons were technical or procedural: one was a pilot participant (Sub00); one was excluded because of a recording failure that left no usable video (Sub07); three were excluded because frame drops during recording left incomplete video sequences (Sub12, Sub26) or an incomplete processed sequence (Sub15); one was excluded because reflections from metal-framed spectacles corrupted the perioral region (Sub13); and one was excluded because the performed movements did not follow the instructed exercises (Sub24). All exclusion decisions were made during data screening, before any model was trained, and were therefore independent of model performance. All recordings were made in a controlled laboratory environment with standardized lighting conditions maintained at an average illuminance of 1,245 lux for all subjects. Approval of all ethical and experimental procedures and protocols was granted by the ethics commission at Ärztekammer des Saarlandes, Saarbrücken, Germany (Identification Number: 13/24) and followed local laws and institutional guidelines. All participants provided written informed consent prior to participation. Data acquisition for each participant was conducted in a single continuous session.

Participants were comfortably seated at a standardized distance from a display monitor in the controlled laboratory environment. They were instructed to watch video stimuli displaying various facial yoga expressions which were designed to activate specific facial muscles and hold each expression for 10 s.

The sequence of five prescribed movements, illustrated in Figure 3, was repeated across subjects to evaluate model specificity and temporal consistency. To correspond with the analysis labels presented later in our results, the sequence consisted of: (1) Fish Mouth 1 (FM1), involving the puckering of lips; (2) EPRA1 (Extraperioral Region Activity: Eyebrows Together), where subjects drew their eyebrows together; (3) Smile (S), targeting the zygomaticus major; (4) EPRA2 (Extraperioral Region Activity: Eyebrows Raised), involving the lifting of both eyebrows; and (5) Fish Mouth 2 (FM2), a repetition of the first task.

**FIGURE 3:**
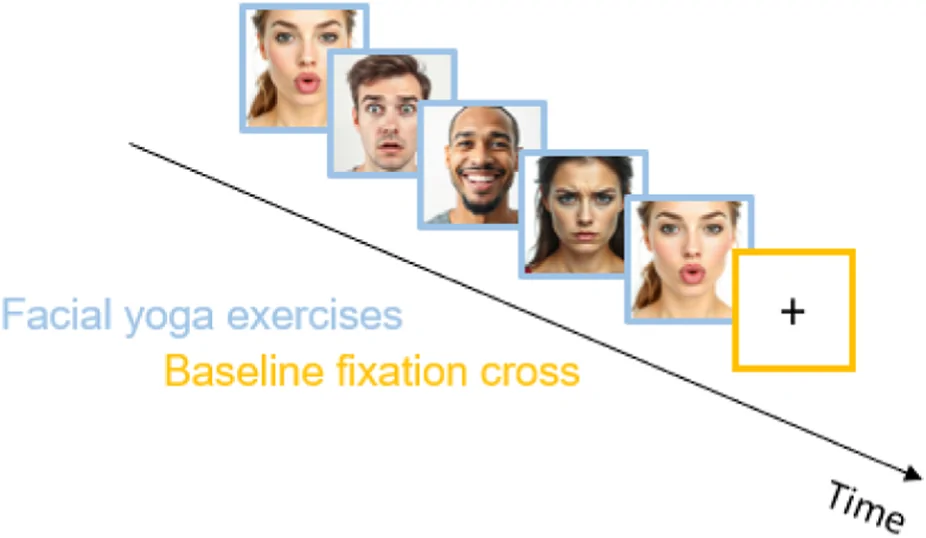
Visual stimuli for facial movement task, showing face yoga exercises displayed on television that participants were instructed to replicate.

The EPRA tasks (EPRA1 and EPRA2) served as negative controls. As reported in Section 4.2, this assumption did not hold for EPRA2. Since the model’s input is cropped to the perioral region, these tasks were designed to assess specificity by evaluating the model’s response to upper-face movements that are mechanically distinct from the target zygomaticus action, provided that no physiological co-activation occurs. Each task sequence was followed by a Baseline recording period (30 s), during which participants were directed to maintain a neutral facial expression and focus on a black fixation cross. This allowed for the capture of resting-state neuromuscular activity.

#### 3.1.2 Visual data acquisition

Video recordings were captured from 23 subjects at 160 fps with 3 millisecond exposure time under controlled laboratory conditions with standardized lighting. Each subject was recorded for 80 s. The high frame rate was selected to capture rapid facial muscle dynamics associated with zygomaticus major activation during facial expressions.

#### 3.1.3 sEMG data acquisition

4-mm Ag/AgCl passive electrodes (bioMed Products, United States) were used to record surface electromyography bilaterally from the left and right zygomaticus major muscles. Electrodes were placed in a bipolar configuration according to standardized facial sEMG guidelines (Fridlund and Cacioppo, 1986). sEMG was recorded with a sampling frequency of 4,800 Hz. All acquired signals were interconnected via biosignal amplifiers (g.tec medical engineering GmbH, Austria). The trigger events were captured with a triggerbox (g.tec medical engineering GmbH, Austria), which was also connected to the amplifiers, to a soundcard (Focusrite Audio Engineering Ltd., England), and to a function generator (Tektronix, United States). The audio of the video stimuli was transmitted through an active near-field loudspeaker (Neumann, Germany) via a soundcard. MATLAB/Simulink R2020b (MathWorks, United States) was used to develop and execute the entire setup.

### 3.2 Data processing

The data processing pipeline was designed to establish temporal synchronization between visual mouth movements and corresponding sEMG amplitude envelopes. This process involved several key steps:

#### 3.2.1 Visual data processing

To improve computational efficiency, the raw video recordings were down-sampled from 160 fps to an effective frame rate of 80 fps by processing every other frame. This approach reduced the computational load while remaining sufficient for capturing the temporal dynamics of macro-expressions (Savchenko, 2023). The visual data processing pipeline then extracted standardized mouth regions from the temporally down-sampled frames. Using MediaPipe (Lugaresi et al., 2019) face mesh, we tracked four facial landmarks surrounding the mouth to define a region of interest (ROI) that encompasses the perioral region. By processing these four facial landmarks across all subjects for all frames, we identified the maximum rectangular mouth ROI dimensions during peak smile expressions, where subjects exhibited maximal mouth stretching. This detection was used to determine a standardized crop size applied uniformly across all subjects, ensuring consistent spatial resolution for perioral region cropping. Crops were stored at 304×168 pixels (width × height) and, at load time, resized to 70% of the stored dimensions using Lanczos resampling, giving a model input resolution of 212×117 pixels. The crop size was determined from the maximum mouth extent observed across all 23 analyzed subjects, including subjects subsequently assigned to test folds; since this is a single spatial dimension that does not depend on the sEMG target signal, we do not consider it a meaningful source of information leakage, but state it here for transparency. Each extracted crop was then converted to grayscale. The zygomaticus major electrodes were positioned on the upper cheek, outside the perioral crop region, and were therefore not visible in the model input.

#### 3.2.2 sEMG signal processing

Based on the works of Strauss et al. (2020), the raw sEMG signals were segmented into epochs (3 s prior to the start of the trial to 3 s past the end of the trial) (Strauss et al., 2020). The segments were notch filtered (50 Hz, 2nd order IIR filter) and band-pass filtered (60 Hz–1,000 Hz, 2000th order FIR filter) (Strauss et al., 2020). The filtered signals were then re-referenced. The signals were baseline-corrected and rescaled to the interval [0,1] by subtracting the minimum RMS amplitude and dividing by the difference between the maximum and minimum RMS amplitude. These extrema were computed independently for each recording, that is, separately for each trial of each participant, so that no normalization parameter was shared across participants or trials and no test-subject information contributed to the normalization of any other recording.

The left and right zygomaticus major envelopes were normalized separately as described above and then averaged sample-wise into a single bilateral envelope, which served as the target signal throughout. ViMG therefore estimates bilateral rather than unilateral zygomaticus major activity.

The resulting target is dimensionless: ViMG estimates a trial-relative proportion of that trial’s amplitude range rather than an absolute value in microvolts, and the reported MAE and RMSE are in normalized units. Since the model outputs a single scalar per sequence, the ground truth for each training sample was defined as the mean RMS envelope amplitude of that specific input window. This formulation converts the continuous sEMG estimation task into a sequence-to-scalar regression problem. Throughout this manuscript, we refer to this target quantity as the window-averaged sEMG envelope amplitude. ViMG does not reconstruct the raw sEMG waveform.

Synchronization between modalities was established through the common trigger recorded by the triggerbox, which was connected to the amplifiers and the soundcard. Following epoching, the RMS envelope was computed using MATLAB’s envelope function with a moving-RMS window of 4,800 samples (1 s), which is centered on each sample, and was subsequently resampled to 80 Hz to match the processed video frame rate. Each envelope sample was then paired index-wise with the corresponding video frame, so that video frame n was aligned with envelope sample n ; no additional temporal offset was applied.

Because the RMS window is centered, the envelope value assigned to a given time point reflects raw sEMG activity in the surrounding ±0.5 s. We did not compensate for acquisition latency or for the electromechanical delay between electrical activation and visible skin deformation, which is typically on the order of tens of milliseconds. Relative to the 0.7-s prediction window and the 1-s envelope smoothing, a delay of this magnitude is small, but we note that it was not measured and constitutes a systematic offset between modalities.

#### 3.2.3 Temporal windowing

To facilitate sequence modeling, the synchronized data streams were segmented into non-overlapping windows of 0.7 s, corresponding to 56 consecutive frames from the video stream. This window size was chosen to balance GPU memory constraints with the physiological requirement of capturing the temporal evolution of macro-expressions, which typically unfold over durations exceeding 500 m (Künecke et al., 2014). For each video sequence, the target label was calculated as the mean value of the 56 corresponding sEMG envelope samples. This window-to-scalar supervision strategy aligns the temporal context of the visual motion patterns with the aggregate muscle activation intensity, providing a stable training signal for the regression task.

#### 3.2.4 Temporal rates and effective resolution

To avoid ambiguity, we distinguish the rates involved. Video was processed at 80 fps, and the sEMG envelope, computed with a 1-s sliding RMS window, was resampled to 80 Hz for alignment. Both streams were then segmented into non-overlapping 0.7 s windows, so the model produces one prediction per 0.7 s ( ≈ 1.43 Hz). Because each prediction summarises 0.7 s and the reference envelope is itself smoothed over 1 s, the effective temporal resolution is approximately 1.7 s; the 80 Hz figure refers to alignment prior to windowing, not to the rate of independent estimates.

### 3.3 Model architecture

The ViMG (Video Myography) model, as shown in Figure 4, uses a hybrid architecture combining a 3D U-Net with a Long Short-Term Memory (LSTM) network for predicting the window-averaged sEMG envelope from video sequences. The model was configured with grayscale video input (1 channel), a single-channel output, 32 initial features, and a dropout rate of p=0.3 for regularization. In total, the model comprises 23,368,994 trainable parameters.

**FIGURE 4:**
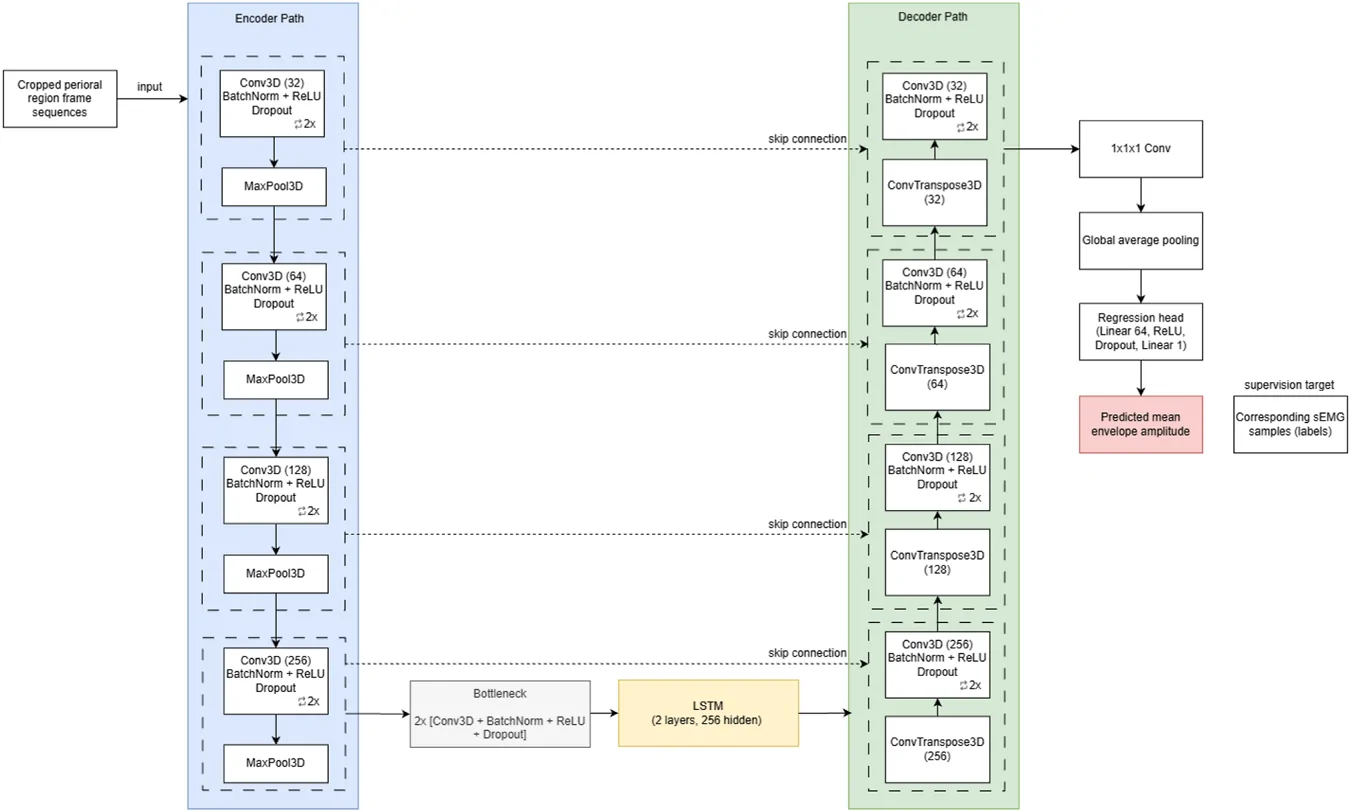
The ViMG (Video Myography) model architecture for sEMG envelope prediction from videos. The diagram illustrates the hybrid network, which integrates a 3D U-Net for spatio-temporal feature extraction with a Long Short-Term Memory (LSTM) layer at the bottleneck intended to capture temporal dependencies.

The 3D U-Net component is structured as an encoder-decoder network. The encoder path processes input sequences of shape (N,1,56,117,212) , where N is the batch size, 1 is the number of channels, 56 is the number of frames (depth), and 117×212 is the spatial resolution of the perioral crop after resizing (Section 3.2.1). Each block is composed of two repetitions of a 3D Convolution, followed by Batch Normalization, a ReLU activation, and Dropout: Conv3D→BatchNorm3d→ReLU→Dropout3d . The 3D convolutional layers use a 3×3×3 kernel with a padding of 1. After each encoder block, a 3D max-pooling operation with a 2×2×2 kernel down-samples the feature maps, progressively increasing the receptive field, while the first convolution of each subsequent block doubles the number of feature channels.

At the bottleneck, the feature tensor has shape (N,512,3,7,13) , corresponding to batch size, channels, and the downsampled depth, height, and width. This tensor is permuted to (N,3,7,13,512) and reshaped to (N,273,512) , so that the depth, height, and width positions are flattened into a single sequence of 3×7×13=273 elements, with the 512 bottleneck channels forming the feature dimension. A two-layer LSTM with a hidden size of 256 processes this sequence, yielding (N,273,256) . The full output sequence is then reshaped to (N,3,7,13,256) and permuted to (N,256,3,7,13) before entering the decoder path.

Because the flattening is ordered as depth, then height, then width, consecutive elements of the sequence correspond predominantly to neighbouring spatial positions within the same temporal slice; the depth index advances only every 7×13=91 elements. The recurrent layer therefore performs a sequential scan over the joint spatio-temporal bottleneck rather than a purely temporal one.

The decoder path symmetrically mirrors the encoder. It consists of four up-sampling blocks, each initiated by a 2×2×2 transposed convolution. The first of these preserves the 256 channels produced by the recurrent layer; each subsequent transposed convolution halves the number of feature channels. Skip connections concatenate each up-sampled decoder feature map with the corresponding encoder feature map; because pooling with floor division yields odd extents at several levels, encoder feature maps are center-cropped to match the decoder resolution before concatenation.

The decoder is retained despite the scalar output so that spatial aggregation is performed on the highest-resolution decoder feature map ( 48×112×208 after cropping), in which bottleneck context has been combined with fine-grained encoder detail through the skip connections, rather than on the coarse 3×7×13 bottleneck directly. This was intended to preserve the subtle perioral deformation cues that distinguish zygomaticus major activation.

At the end, a 1×1×1 convolutional layer maps the last decoder block to a single channel, and global average pooling reduces this volume to one value per sequence. A small regression head applied to this scalar (Linear 1 to 64, ReLU, dropout, Linear 64 to 1) produces the predicted mean sEMG envelope value. The contributions of the recurrent layer and of this aggregation strategy are examined through ablation and are discussed in the “Ablations and Learned Baseline” section.

### 3.4 Training

#### 3.4.1 Experimental setup

The proposed model was implemented in PyTorch (Paszke et al., 2019) and trained on a single NVIDIA GeForce RTX 4090 GPU with 24 GB of VRAM. To ensure full reproducibility, the training process was executed under a deterministic configuration. This involved setting a global random seed of 42 for relevant libraries (e.g., NumPy, PyTorch) and configuring cuDNN to use deterministic algorithms by disabling its non-deterministic benchmark mode.

Input frames were converted to grayscale, resized to 70% of the stored perioral crop dimensions using Lanczos resampling, and scaled to the interval [0,1] by division by 255. No per-image or per-dataset mean-variance standardization was applied, and no data augmentation was used at any stage of training.

#### 3.4.2 Optimizer and model selection

We trained the model using the Adam optimizer (Kingma and Ba, 2014) with an initial learning rate of $1\times 10^{-4}$ , a weight decay of $5\times 10^{-4}$ , and a batch size of 2. Training ran for a maximum of 250 epochs. A ReduceLROnPlateau scheduler reduced the learning rate by a factor of 0.6 whenever the validation mean squared error failed to improve for 10 epochs, and early stopping was applied with a patience of 40 epochs and a minimum improvement threshold of 0. The checkpoint with the lowest validation mean squared error was retained for test evaluation. Model selection used the unweighted validation MSE rather than the weighted training objective, because the weighting concentrates the loss on a small number of high-amplitude windows and made it an unstable selection criterion on a validation set of four subjects. The training objective itself is detailed in the following subsection.

#### 3.4.3 Custom weighted loss function

To accommodate the signal dynamics of facial sEMG, in which high-amplitude readings reflect substantial facial muscle activation (particularly during movement transitions like smile onset), whereas low-amplitude signals approaching zero denote minimal or absent facial activity, we implement a weighted Mean Squared Error (MSE) loss function. Our weighting scheme applies a larger penalty to errors when predicting sEMG envelope values above a threshold τ=0.1 , ensuring better capture of these periods of muscle activation. The weighted MSE loss function is defined as:

$$L\left(\hat{y},y\right)=\frac{1}{n}\overset{n}{\underset{i=1}{\sum }}w_{i}{\left({\hat{y}}_{i}-y_{i}\right)}^{2}$$ (1)

where n∈ N, the weights $w_{i}$ are determined by the reference value $y_{i}$ relative to the threshold τ :

$$w_{i}=\left\{\begin{array}{cc} 15 & \text{if }y_{i}>\tau \\ 1 & \text{otherwise.} \end{array}\right.$$ (2)

Equation 1 represents the weighted Mean Squared Error (MSE) loss function, where ${\hat{y}}_{i}$ is the model’s predicted mean sEMG envelope value for the i -th input sequence, $y_{i}$ is the corresponding target sEMG envelope value, n is the number of sequences, and $w_{i}$ is the weight factor. The weight factor $w_{i}$ , defined in Equation 2, applies a value of 15 if the target value $y_{i}$ exceeds the threshold τ=0.1 , while otherwise applying a unit weight. The weighting was motivated by the sparsity of significant sEMG activity in the target signal. We note that the ablation reported in Section 4.4 (Table 1) does not support this design choice: an unweighted objective achieved comparable correlation and lower error. We report the weighted objective here because it was used to train the model described throughout, but we do not claim it is necessary. While a static weight of this magnitude increases the penalty for missing active regions, we acknowledge that it could theoretically lead to overfitting high-amplitude noise artifacts. We mitigated this risk by incorporating 3D spatial dropout (p=0.3) in every convolutional block of the U-Net, including the bottleneck and decoder, and standard dropout in the regression head, to prevent co-adaptation of features (Hinton et al., 2012), applying L2 weight decay $\left(5\times 10^{-4}\right)$ to constrain model complexity, and using early stopping based on validation MSE. Our subject-independent cross-validation protocol ensures that the reported performance reflects the model’s ability to generalize anatomical deformation patterns rather than memorizing subject-specific sensor noise.

**TABLE 1:** Ablation studies and learned baseline. All variants use the same folds, the same subject-independent train/validation/test partitioning, and the same model-selection procedure as the full model. F1–F5 denote the five cross-validation folds.

Model	Metric	F1	F2	F3	F4	F5	Mean (SD)
Full ViMG	MAE	0.241	0.186	0.145	0.198	0.198	0.194 (0.034)
	RMSE	0.263	0.208	0.180	0.236	0.262	0.230 (0.036)
	r	0.872	0.632	0.630	0.536	0.634	0.661 (0.125)
No LSTM (spatial only)	MAE	0.147	0.191	0.142	0.180	0.192	0.171 (0.024)
	RMSE	0.176	0.209	0.171	0.201	0.254	0.202 (0.033)
	r	0.868	0.640	0.647	0.601	0.628	0.677 (0.108)
Unweighted loss (1:1)	MAE	0.062	0.101	0.085	0.117	0.100	0.093 (0.020)
	RMSE	0.108	0.156	0.147	0.187	0.215	0.163 (0.041)
	r	0.841	0.698	0.618	0.583	0.625	0.673 (0.103)
Landmark baseline	MAE	0.108	0.154	0.124	0.137	0.138	0.132 (0.017)
	RMSE	0.143	0.181	0.150	0.186	0.196	0.171 (0.023)
	r	0.747	0.671	0.759	0.617	0.556	0.670 (0.086)

This weighting strategy addresses the imbalanced distribution of target values in facial sEMG envelope data, where the zygomaticus major muscle exhibits predominantly quiescent periods (near-zero activation) punctuated by brief, significant activity during expressions. By assigning greater importance to samples with high sEMG envelope values, the loss function incentivizes the model to accurately predict these high-amplitude activations, preventing it from being biased toward the more frequent low-activity baseline.

#### 3.4.4 Batching and evaluation procedure

We used a batch size of 2 during model training, a limit necessitated by GPU memory constraints when processing high-resolution 3D spatiotemporal tensors. Each batch consisted of 56-frame video sequences paired with their corresponding ground-truth sEMG targets. Within each training step, the model’s 3D output was reduced by averaging over depth, height, and width to produce a single scalar envelope prediction for each sequence. Gradients were computed via backpropagation using a custom weighted MSE loss, followed by an optimization step using the Adam optimizer. To evaluate model performance, we computed the Pearson correlation coefficient between the predicted scalar outputs and the ground-truth mean RMS envelope amplitudes for each synchronized 0.7-s temporal window. This approach assesses the model’s ability to track the average neuromuscular intensity across the full task duration. Metrics were aggregated across all cross-validation folds to provide an estimate of generalizability. Detailed plots comparing predicted and reference sEMG envelopes are provided in the results section and supplementary material.

#### 3.4.5 Cross-validation strategy

We evaluated the model using subject-level five-fold cross-validation across the 23 participants. Test folds were assigned by scikit-learn’s (Pedregosa et al., 2011) KFold (shuffle enabled, seed 42), giving test sets of 5, 5, 5, 4 and 4 subjects. Within each fold, four of the remaining subjects were held out as a subject-independent validation set, chosen deterministically from a fold-specific seed, leaving 14 or 15 subjects for training. Learning-rate scheduling, early stopping, and checkpoint selection were driven exclusively by the validation mean squared error; the test fold was evaluated once, after training, using the validation-selected checkpoint. Each participant was held out for testing exactly once. Fold composition is recorded for every run.

#### 3.4.6 Spatial attention map generation

To spatially resolve the predicted sEMG envelope and identify its muscular origins within the video frames, we employed a gradient-based saliency method (Simonyan et al., 2013). This technique generates spatial attention maps, which visualize the pixel-level contributions to the model’s final prediction (Simonyan et al., 2013; Zeiler and Fergus, 2014; Springenberg et al., 2014). The process was conducted on the test set subjects from each cross-validation test fold using the corresponding trained model. The procedure begins by preparing the input video data. For each subject, the full sequence of mouth region frames was loaded, resized to 70% of its original dimensions using Lanczos (Duchon, 1979) resampling, and converted to grayscale tensors. The analysis was performed by processing these frames in non-overlapping windows of 56, consistent with the input sequence length used during training. For each 56-frame window, an attention map was generated through a multi-step process. First, the input window was passed through the trained model to generate a prediction. Next, with gradient computation enabled for the input tensor, a backward pass was executed from the output to compute the gradient of the model’s prediction with respect to the input pixels. The resulting gradient for each pixel quantifies the sensitivity of the output to a change in that pixel’s intensity. The saliency map was then formulated by taking the absolute value of these gradients, capturing the magnitude of influence for each pixel. Since the input is grayscale, this resulted in a 2D saliency map for each of the 56 frames in the window. This process was repeated for all windows within a subject’s video sequence. The outcome of this procedure is a series of qualitative heatmaps, one for each video frame. These maps illustrate how the model localizes its focus within the perioral region to predict muscle activation patterns. A qualitative interpretation of these maps is presented in the Results and Discussion section.

#### 3.4.7 Levels of analysis and statistical procedure

We distinguish four levels of analysis. At the window level, the model produces one predicted value per 0.7-s window, paired with the corresponding reference envelope mean. At the subject level, a Pearson correlation is computed between predicted and reference values across all windows of that subject, evaluated within the fold in which the subject was held out; each subject therefore contributes exactly one coefficient. At the fold level, the reported value is the arithmetic mean of the subject-level correlations of that fold’s test subjects. At the overall level, the reported mean is the mean of the subject-level correlations across all subjects.

Because windows within a subject are temporally adjacent and the reference envelope is smoothed over 1 s, window-level observations are not statistically independent. Window-level correlations are therefore treated as descriptive summaries of within-subject tracking, and all inferential statistics are computed at the subject level, where subjects constitute independent observations.

For inference, subject-level correlations were Fisher z -transformed and a one-sample t -test applied against a null hypothesis of zero correlation, that is, that predicted and reference envelopes are uncorrelated within subjects. The 95% confidence interval was computed in z -space as $\bar{z}\pm t_{0.975,22}\;SE_{\bar{z}}$ and back-transformed. Reported correlation means are given untransformed for interpretability, while inference was performed in z -space.

### 3.5 Landmark-based baseline

To assess whether the proposed architecture captures information beyond relatively simple perioral kinematics, we trained a learned baseline operating on facial landmarks rather than pixels. The four MediaPipe landmarks used to define the perioral crop (indices 136, 365, 167, and 411) were extracted for every frame during preprocessing. From these we derived 22 features per frame: the eight landmark coordinates, the six pairwise inter-landmark distances, and the eight frame-to-frame coordinate displacements. Features were standardised within subject. Each 56-frame window of features was passed to a single-layer GRU with a hidden size of 64, followed by a two-layer regression head (Linear 64 to 32, ReLU, Linear 32 to 1), giving 19,009 trainable parameters.

The baseline was trained and evaluated under exactly the protocol used for the full model: the same subject-level five-fold partitioning, the same train/validation/test split within each fold, the same weighted MSE objective, and model selection on validation MSE with early stopping. It is reported alongside the ablations in Section 4.4 and Table 1.

## 4 Results and discussion

### 4.1 Aggregate performance analysis across all folds

Table 2 presents the mean correlation for each fold and the overall mean across folds. Across all subjects, the analysis revealed a positive correlation between the predicted and reference normalized mean sEMG envelopes (left and right zygomaticus major muscle). The model achieved a mean Pearson correlation coefficient across folds of 0.661, with a subject-level arithmetic mean of r=0.667 (SD 0.412). Averaging the subject-level correlations in Fisher z space and back-transforming gives r=0.83 , 95% CI [0.681,0.909] . On an individual level, a large effect size was observed for 18 of the 23 participants.

**TABLE 2:** Overall performance metrics for sEMG envelope estimation across 5-fold cross-validation.

Folds	Subjects (n)	MAE	RMSE	Pearson’s r
Fold 1	5	0.241	0.263	0.872
Fold 2	5	0.186	0.208	0.632
Fold 3	5	0.145	0.180	0.630
Fold 4	4	0.198	0.236	0.536
Fold 5	4	0.198	0.262	0.634
**Mean across folds**	**23**	**0.194**	**0.230**	**0.661**
Landmark baseline (LB)	23	0.132	0.171	0.670

Subject-level arithmetic mean r=0.667 (SD 0.412). Mean of Fisher z -transformed subject-level correlations, back-transformed: r=0.83 , 95% CI [0.681,0.909] . Effect sizes (L/M/S): 18/1/4. One-sample t -test on Fisher z -transformed subject-level correlations against zero: t(22)=7.078 , p<0.001 .

Bold values indicate the mean across folds.

When analyzed per fold, the mean Pearson correlation varied from r=0.536 to r=0.872 . Inspection of the per-subject results indicates that this variance is driven by a small number of subjects with negative correlations (Subjects 09, 14, 18 and 27) rather than by uniformly weaker folds. In Subjects 18 and 27, spontaneous facial movements created sharp sEMG envelope peaks that lacked clear visual correlates, leading to a mismatch between the visual input and the ground truth. Subject 14 was recorded with an anomalous frame count (9,189 frames against 6,580–7,094 for all other subjects), which may reflect an acquisition error and compromise its alignment with the sEMG time base. Subject identifiers refer to original enrollment IDs from the initial cohort of 30. Individual prediction versus reference signal plots for each subject are provided in the supplementary material.

Figure 5 shows predicted against reference values for three subjects. Predictions scale with the reference but at a slope substantially below unity (mean slope 0.42 across subjects, SD 0.43; 21 of 23 subjects below 1), indicating that ViMG recovers the relative time course of the envelope while compressing its amplitude range.

**FIGURE 5:**
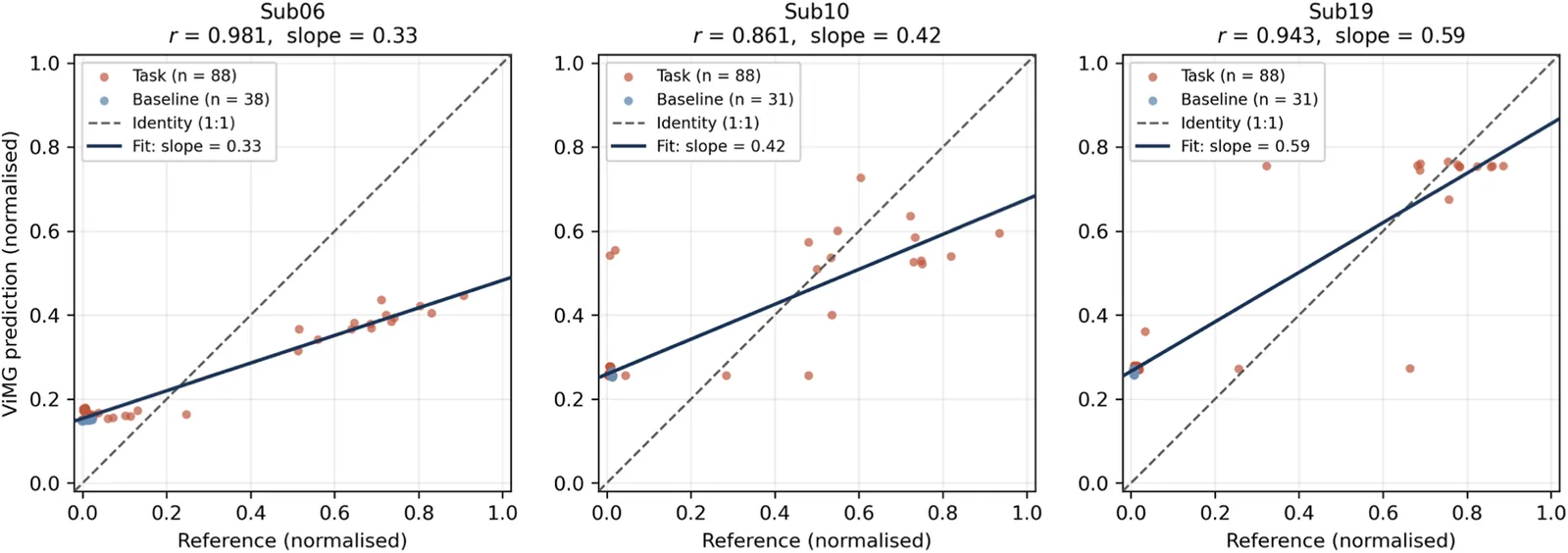
Predicted against reference window-averaged sEMG envelope for three subjects, with task and baseline windows shown separately. Dashed line, identity; solid line, least-squares fit. Predictions scale with the reference at a slope below unity in all three cases. Scatter plots for all 23 subjects are provided in the supplementary material.

### 4.2 Task-specific performance and model specificity

Figure 6 illustrates the task-specific correlation distributions across all test subjects (n=23) for the prescribed facial movements. This analysis evaluates the model’s ability to distinguish zygomaticus major activity from other facial actions.

**FIGURE 6:**
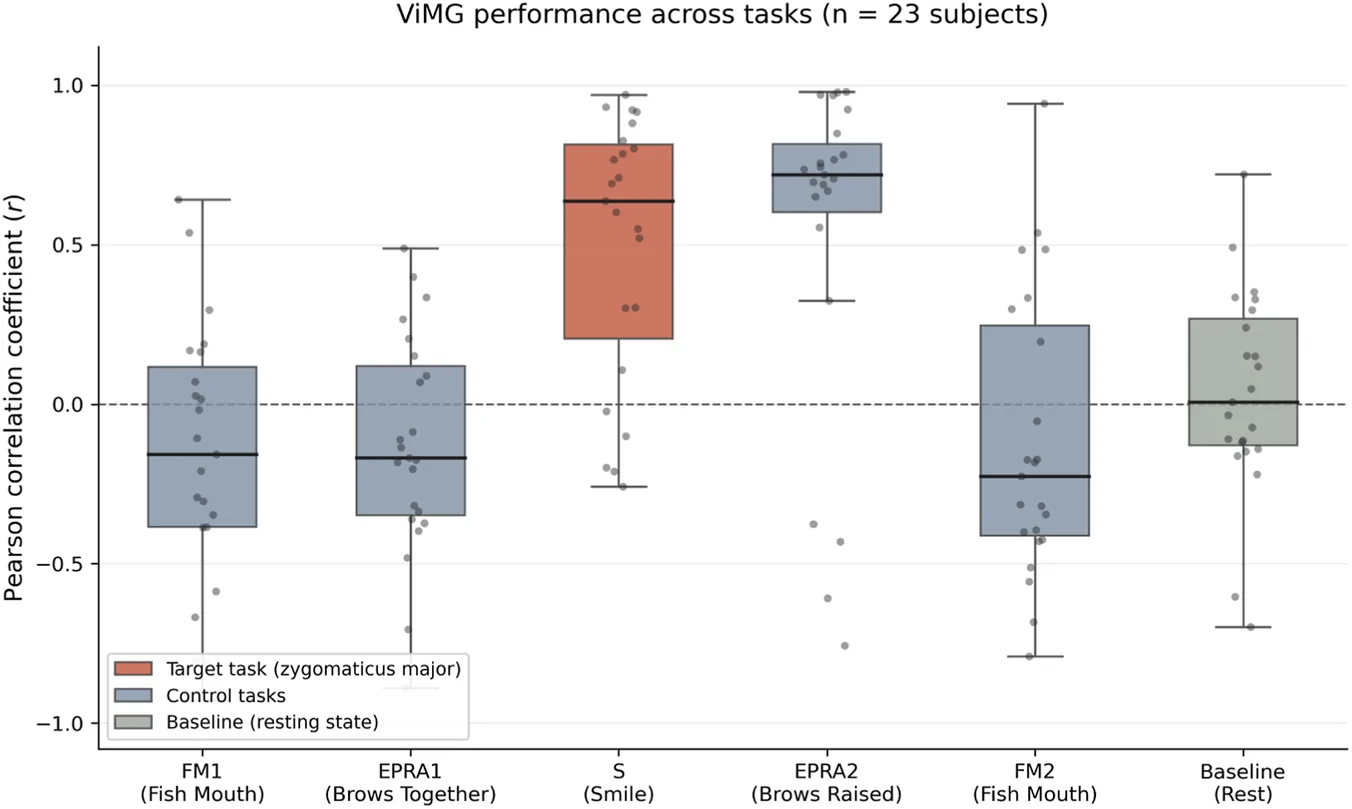
Task-wise Pearson correlation distributions across all 23 subjects. Box colours indicate the role of each task in the experimental design: the target zygomaticus major task (Smile), the control tasks (FM1, EPRA1, EPRA2, FM2), and the resting Baseline. Abbreviations: FM1/FM2 (Fish Mouth repetitions), EPRA1 (Eyebrows Together), S (Smile), EPRA2 (Eyebrows Raised), Baseline (resting state).

As visualized in Figure 6, the model demonstrates distinct response patterns across tasks. The target task Smile (S) achieves a median correlation of r=0.64 (mean r=0.50 ). The EPRA2 (Eyebrows Raised) task, introduced as a negative control, yielded a comparable or higher median correlation (median r=0.72 , mean r=0.54 ). Post-hoc inspection of the reference sEMG revealed non-negligible activity in the zygomaticus major channel during this task (mean amplitude ≈18% of maximal smile), so the model’s predictions tracked a reference signal that was itself active rather than responding to a silent target. The origin of that activity cannot be determined from the present data. Plausible explanations include genuine co-activation of the zygomaticus major during high-effort facial posturing, a form of motor overflow described in the literature (Tassinary et al., 2007); mechanically coupled deformation propagating from the upper face to the perioral region; electrical crosstalk from adjacent active muscles into the zygomaticus electrode pair; or visual features associated with the task that covary with the reference signal without reflecting zygomaticus activity. Distinguishing between these accounts would require additional measurements, such as multi-site sEMG or independent kinematic tracking, and we therefore report the EPRA2 result as an observation that qualifies the interpretation of this condition as a negative control rather than as evidence of a specific physiological mechanism.

Similarly, the Fish Mouth (FM1 and FM2) tasks exhibited negative median correlations ( r=−0.16 and r=−0.23 ), though with notable inter-subject variability. This variance is attributable to the mechanical coupling at the modiolus, where the target zygomaticus major and the active Orbicularis oris converge. While the task primarily targets the lips, intense puckering can mechanically deform the cheek or induce involuntary co-contraction in some subjects, leading to genuine but non-target signal correlations (Komisarek et al., 2025). Furthermore, because the zygomaticus signal amplitude during this task is inherently low, the signal-to-noise ratio (SNR) is reduced; in low-SNR regimes, Pearson correlation becomes sensitive to minor fluctuations, resulting in the observed statistical spread. Despite this, the median correlation for the target Smile task (r=0.64) is clearly separated from that of the Fish Mouth controls ( r=−0.16 and r=−0.23 ), which is consistent with selectivity of the model for the target action. The EPRA2 control, however, yielded a median correlation exceeding that of the Smile task, so the present data do not establish selectivity in general. No formal statistical comparison between task conditions was performed, and these observations are therefore descriptive.

### 4.3 Model interpretability via attribution mapping

To provide interpretability for the model’s predictions, we generated gradient-based attribution maps and averaged the attributions across all frames for each subject for the entire length of the video sequence. Figure 7 displays these averaged maps overlaid on a starting frame for the top two performing subjects from each of the five cross-validation test folds. Visual inspection of these maps reveals a consistent pattern: the model’s attention is predominantly focused on anatomically relevant perioral regions. In particular, high-intensity attribution is concentrated around the edges of the mouth and the nasolabial folds, which correspond to the known anatomical location and area of influence of the zygomaticus major muscle. Conversely, attribution values are notably lower in the lateral regions corresponding to the buccinator muscle belly. This pattern is consistent with the model relying on anatomically plausible perioral regions associated with zygomaticus major action, particularly the nasolabial fold, rather than on lateral cheek regions. We note that a gradient-based attribution map indicates which pixels influence the model output; it does not establish that the model has isolated a specific muscle or its contraction direction. The model’s strong focus on the deformation of the nasolabial folds (smile lines) suggests it is more than just a simple teeth detector. It also suggests that the model is responding to the physiological mechanics of the smile itself, which are defined by skin movement, rather than relying on a single feature like visible teeth. This focus is particularly clear in subjects such as Subject 20 (r=0.98) and Subject 01 (r=0.95) , where the attribution is tightly localized to the lip contour and nasolabial region. While these maps highlight anatomically plausible features, we also observed high attribution intensity along the map borders for a subset of subjects. In other cases the attribution was more diffuse, as seen in Subject 06 (r=0.98) , or more scattered, as in Subject 19 (r=0.94) . Nevertheless, the general trend holds across the majority of high-performing subjects. This qualitative analysis indicates that the model’s predictions are informed by anatomically plausible perioral regions, consistent with a mapping between perioral kinematics and the zygomaticus major envelope, although saliency analysis alone cannot confirm such a mapping.

**FIGURE 7:**
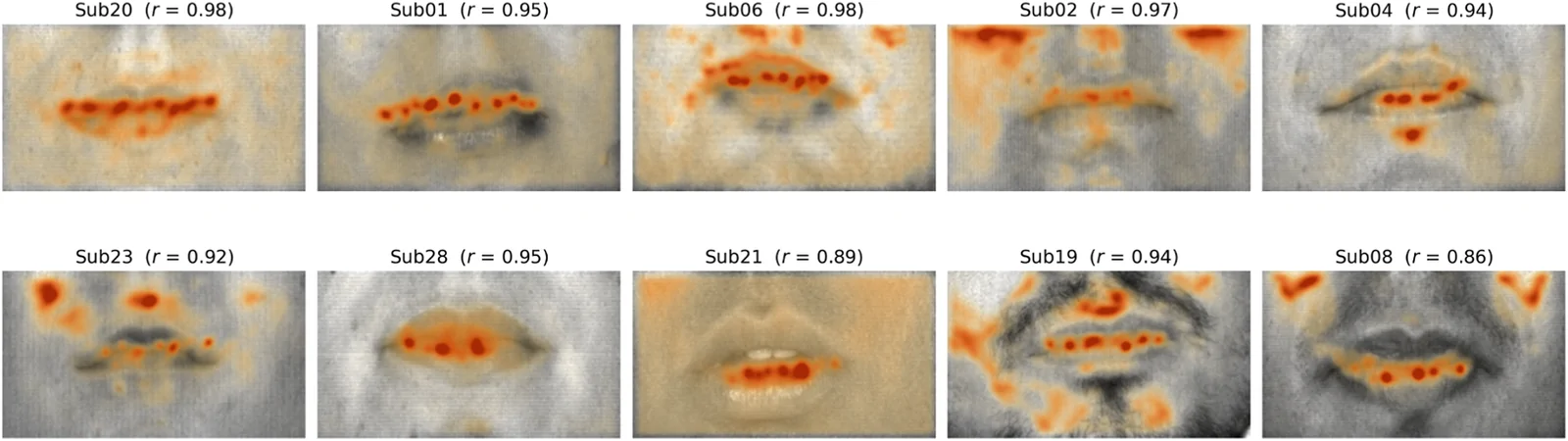
Mean spatial attribution maps, averaged across all frames, showing which perioral regions influence the model output. The subjects shown are the two highest-performing subjects from each cross-validation fold and therefore represent favourable rather than typical cases.

### 4.4 Ablations and Learned Baseline

To quantify the contribution of the recurrent layer, we conducted an ablation in which the LSTM was replaced by a position-wise projection (a linear layer followed by ReLU) of the same output dimensionality, leaving the encoder, decoder, and skip connections unchanged. As shown in Table 1, this did not reduce performance: the mean fold-level correlation was 0.677 without the recurrent layer against 0.661 with it, with lower MAE and RMSE. A second ablation replacing the weighted objective with an unweighted MSE gave a mean correlation of 0.673, again with lower error. Across all variants, including the landmark baseline, mean correlations fall between 0.661 and 0.677, a range well within the fold-level standard deviations (0.09–0.13). We therefore find no evidence that either the recurrent layer or the loss weighting contributes to performance on this task, and we do not claim that they do.

This pattern was consistent across folds rather than driven by individual folds. We note that with only four or five test subjects per fold, fold-level means are strongly influenced by individual subject variance, and we therefore base our interpretation on the aggregate across folds.

The landmark-based baseline achieved a mean subject-level correlation of 0.670 (fold mean 0.670, SD 0.086), with MAE 0.132 and RMSE 0.171. This matches the full model on correlation and exceeds it on both error metrics, with roughly three orders of magnitude fewer parameters. The baseline was also more consistent across subjects: its lowest per-subject correlation was −0.20 , against four subjects below zero for the full model. We conclude that much of the recoverable signal is explained by coarse perioral kinematics, and that the present results do not demonstrate an advantage for pixel-level spatiotemporal modelling on this task.

### 4.5 Discussion

#### 4.5.1 Performance comparison and benchmarking

Our contactless approach for estimating window-averaged sEMG envelope amplitude achieved a mean Pearson correlation coefficient of r=0.661 across all folds. Performance on the target “Smile” task was lower than this aggregate (median r=0.64 , mean r=0.50 ), and the mean subject-level correlation on task windows alone was r=0.638 against 0.667 on all windows, indicating that the extended baseline periods contribute modestly to the aggregate. We note that the aggregate correlation does not by itself establish performance during baseline periods, and we make no claim about baseline-specific accuracy on that basis.

Perusquia-Hernandez et al. (2020) reported accuracy rates of 81% for AU06 (Orbicularis Oculi) and 82% for AU12 (zygomaticus major) detection using their multi-modal sEMG and computer vision approach, with Cohen’s Kappa values of 0.49 and 0.53, respectively. While direct comparison is challenging due to different evaluation metrics and tasks, our mean correlation coefficient of 0.661 suggests that contactless sEMG envelope prediction on short temporal windows can correlate with actual muscle activity patterns, although as reported in Section 4.4 this level of performance did not require the proposed architecture.

Contact-based sEMG systems typically achieve higher precision due to direct electrode placement on the skin surface. However, long-term monitoring can cause subject discomfort and may lead to unnatural or forced facial expressions, thereby compromising the recording. Our approach trades some measurement precision for contactless operation, which eliminates electrode placement procedures, reduces user discomfort, and enables more naturalistic facial expression analysis.

We emphasize that ViMG does not measure electrical muscle activity. Surface electromyography records the electrical manifestation of motor unit recruitment, whereas ViMG infers an sEMG-derived amplitude measure from its mechanical consequences, namely visible perioral skin deformation. These quantities are physiologically related but not identical, and ViMG is therefore best understood as estimating a visually inferred correlate of the processed sEMG envelope rather than as a substitute for electromyographic measurement.

#### 4.5.2 Architectural effectiveness and design choices

We adopted a hybrid 3D U-Net and LSTM architecture on the expectation that joint spatiotemporal modelling would benefit sEMG envelope estimation, following Yu et al. (2018). The ablations reported in Section 4.4 do not support this expectation for the present task: removing the recurrent layer did not reduce performance, and a landmark-based model with three orders of magnitude fewer parameters performed comparably. We therefore describe the architecture as one workable approach to the task rather than as a demonstrated requirement for it.

A key design choice in this architecture is the aggregation of a 56-frame sequence into a single predictive value. This approach represents a trade-off: while it sacrifices fine-grained temporal precision within the 0.7-s window, it allows the model to learn a more generalized mapping by focusing on the overall envelope of the facial movement. By summarizing the activity over a sequence, the model becomes less sensitive to spurious frame-to-frame visual noise and is better able to capture the complete dynamic of a macro-expression, which aligns well with the smooth, envelope-like nature of the processed sEMG data it aims to predict.

The weighted MSE loss function was intended to emphasize prediction accuracy during periods of significant muscle activity (normalized sEMG envelope >0.1 ) while maintaining reasonable performance during minimal activity periods. This weighting strategy addresses the inherent challenge in sEMG envelope prediction where periods of activation are brief within longer sequences of minimal muscle activity. As detailed in Section 3.2.4, the model produces one estimate per 0.7 s window, derived from an envelope smoothed over 1 s. This resolution is appropriate for tracking the aggregate intensity of macro-expressions, which unfold over durations exceeding 500 m, but is substantially coarser than that of conventional sEMG and does not support analysis of events at the timescale of micro-expressions.

To ensure that the ViMG model was not treated as a complete black box, we included an interpretability pipeline using saliency maps to visualize the model’s focus. Figure 7 shows the averaged saliency maps, which highlight perioral regions corresponding to muscle activation. However, for a subset of subjects, we observed high-intensity attribution values along the map borders. We hypothesize that this is a padding artifact, where the model has learned to associate the artificial edges created by zero-padding in convolutional layers with specific predictions. This finding suggests that while the model learns task-relevant features, its explanations can be influenced by such low-level data properties, a common phenomenon in CNN-based saliency analysis.

#### 4.5.3 Comparison with traditional sEMG and computer vision approaches

Our contactless approach introduces advantages over traditional methods while acknowledging current operational trade-offs. Compared to contact-based sEMG, this method inherently bypasses the requirements for physical electrode attachment and skin preparation. This could enable future applications in rapid deployment or long-term monitoring where physical contact is impractical. The absence of facial sensors eliminates tactile feedback that might otherwise influence expression formation, offering a less intrusive alternative for physiological assessment.

When compared to pure computer vision approaches for facial expression recognition, our method provides additional physiological grounding by predicting an sEMG-derived correlate, rather than relying solely on visual feature classification. This physiological grounding provides a more direct and quantitative measure of facial action, enabling a finer-grained analysis than what is possible with classification based on visual features alone.

Beyond these methodological comparisons, it is useful to distinguish two forms of signal mixing. Traditional sEMG is subject to electrical crosstalk, in which electrodes capture volume-conducted potentials generated by neighbouring muscles. Because ViMG tracks mechanical skin deformation rather than electrical potentials, it is not affected by volume conduction. This does not, however, imply that the activity of a single muscle has been isolated: facial muscles and the surrounding soft tissue are mechanically coupled, adjacent muscles can genuinely co-activate, and deformation produced at one site can propagate to another, for example through the shared insertion at the modiolus. A vision-based approach therefore exchanges electrical crosstalk for mechanical coupling effects rather than eliminating signal mixing altogether. Disentangling passive mechanical pull from active contraction of the target muscle remains an open problem for vision-based estimation.

#### 4.5.4 Clinical and applied implications

The contactless nature of our approach may be applicable in clinical settings where traditional sEMG placement may be challenging or contraindicated. For patients with facial injuries, sensitivity to adhesives, or conditions that require extended monitoring, our video-based approach could provide valuable muscle activity assessment without physical contact. The method’s capability for remote monitoring could support telemedicine applications and home-based rehabilitation programs. The absence of electrodes is also relevant in pediatric populations, where the reduced scale of facial anatomy makes standardized placement harder to achieve and increases the likelihood that an electrode pair registers volume-conducted activity from adjacent mimetic muscles, as noted in the Introduction.

In human-computer interaction contexts, our approach could enable more natural and unobtrusive emotion recognition systems that do not require users to wear sensors or undergo electrode placement procedures. This could be particularly valuable for applications in gaming, virtual reality, or assistive technologies where unobtrusive sensing is required.

## 5 Limitations

Several limitations warrant consideration. Our study’s cohort of 23 young adults limits generalizability across age groups and diverse facial anatomies. While the head fixation device was essential for maintaining consistent facial landmark tracking and minimizing motion artifacts that would otherwise compromise the video-sEMG alignment, we acknowledge that this represents a controlled laboratory setting that differs from fully unconstrained natural environments. Relaxing this constraint is a tractable engineering problem rather than a fundamental barrier, and we outline candidate approaches in Section 6.

Video-based estimation depends on the visibility of skin surface deformation, and dense facial hair could in principle obscure the luminance changes on which the method relies. We did not observe a systematic relationship between facial hair and per-subject performance in this cohort, but with few affected subjects these data cannot resolve the question, and we note it as an untested source of variability. Depth sensing or thermal imaging, which are less sensitive to hair occlusion than RGB video, could address this. Facial hair was not an *a priori* exclusion criterion; excluding subjects after observing their performance would have inflated the reported correlations, so all subjects were retained.

Data collection under controlled laboratory conditions with standardized lighting may not reflect performance in naturalistic settings where varying illumination, camera angles, and environmental factors could impact predictions. Our focus on the zygomaticus major muscle constrains applicability to multi-muscle facial expression analysis involving multiple muscle groups. Technical constraints include the processing architecture itself. To produce a single predictive value, the model aggregates a sequence of 56 frames (approx. 0.7 s). This aggregation prevents the model from resolving the precise timing of muscular events within each window, instead providing a summary of the overall mean envelope activity during that period. The limitation is compounded by the 1-s RMS smoothing applied to the reference signal, so that transient events such as the onset of a startle response, subtle muscle tremors, or micro-expressions occurring in under 200 m are not resolvable in principle rather than merely difficult to detect. The fixed perioral crop dimensions were also a limitation, as they do not account for variations in subject head size or camera distance, introducing scale inconsistencies. Lastly, one important limitation of video-based sEMG prediction stems from the fundamental temporal mismatch between electrical and mechanical muscle activity. sEMG sensors can detect pre-kinematic muscle activation, electrical activity that precedes visible facial movement, while video-based prediction is inherently constrained to observable kinematic changes. This temporal lag between electromyographic onset and visible muscle contraction represents a fundamental limitation of vision-based sEMG prediction, as early-phase muscle recruitment occurring below the threshold of detectable movement cannot be inferred from visual data alone.

Our empirical comparison includes a learned landmark-based baseline, which achieved performance comparable to the full model. While the present results show that contactless envelope estimation is possible, they do not establish that a high-capacity spatiotemporal architecture is required for it. Determining which aspects of the task, if any, benefit from such capacity remains an open question.

## 6 Future work

Future work should prioritize: (1) expanding validation to diverse populations across age, ethnicity, and anatomical variations, ensuring the inclusion of subjects with facial hair to test model generalizability; (2) extending the framework to multiple facial muscles for improved expression analysis; (3) refining the input strategy by replacing the fixed crop with a subject-specific dynamic crop to prevent the inclusion of non-target facial areas; and (4) validating the model against spontaneous expressions in natural settings; (5) Removing the head fixation requirement, for example by using the extracted facial landmarks to register each frame to a canonical frontal view prior to ROI extraction, combined with geometric augmentation and, for out-of-plane rotation, depth-aware or multi-camera acquisition. (6) Verifying robustness to the presence of recording hardware in the field of view, for example by inpainting electrode and adhesive regions in the input frames and comparing performance against the unmodified data. (7) Implementing a multi-camera setup to capture 3D facial geometry could improve muscle activity localization by providing depth information, reducing occlusion errors, and enabling a more accurate analysis of individual, overlapping muscles.

Additional research directions include integrating complementary modalities (depth, thermal imaging), applying transfer learning for population adaptation, and exploring clinical applications in facial rehabilitation and neuromuscular disorder detection.

## 7 Conclusion

Our findings demonstrate that ViMG can estimate the window-averaged amplitude of the facial sEMG envelope from video data, achieving an overall mean Pearson correlation of r=0.661 across five folds. For the target Smile action, the median correlation was r=0.64 . A landmark-based model with 19,009 parameters, three orders of magnitude fewer than ViMG, achieved comparable performance, indicating that much of the recoverable signal is explained by coarse perioral kinematics. Gradient-based saliency mapping indicates that the model’s predictions are informed by anatomically plausible perioral regions, particularly mouth corners and nasolabial folds.

Rather than serving as a replacement for clinical electromyography, these results position ViMG as a proof-of-concept for contactless assessment within controlled environments. This non-invasive approach is relevant to cognitive and developmental research, where physical sensors can confound the behaviour under study, so removing them helps preserve ecological validity. By removing the sensory distress associated with contact electrodes, this framework could support monitoring of naturalistic facial affect in vulnerable populations, such as infants or individuals with neurodevelopmental disorders, facilitating more authentic observations in human-computer interaction and affective computing.

## Data Availability

The datasets presented in this article are not readily available because the dataset comprises facial video recordings and synchronized surface electromyography from human participants and therefore contains potentially identifiable information. The data are not publicly available due to institutional data-protection policies and privacy restrictions governing sensitive participant information. In addition, the informed consent obtained from participants does not permit sharing of their data beyond the research team. As the trained models are derived from this confidential data, they are likewise not available. Requests to access the datasets should be directed to FN, nizamani@snn-unit.de.
