# Supplementary material for: ViMG: a contactless deep learning framework for estimating sEMG amplitude envelopes from facial videos
Source: Front Bioeng Biotechnol. 2026 Oct 30;14:1945313. doi: 10.3389/fbioe.2026.1945313 (PMC13760531; doi:10.3389/fbioe.2026.1945313)
Supplement: Supplementary file 1 [file DataSheet1.zip › results/full_model/figures/figure5_taskwise.pdf]

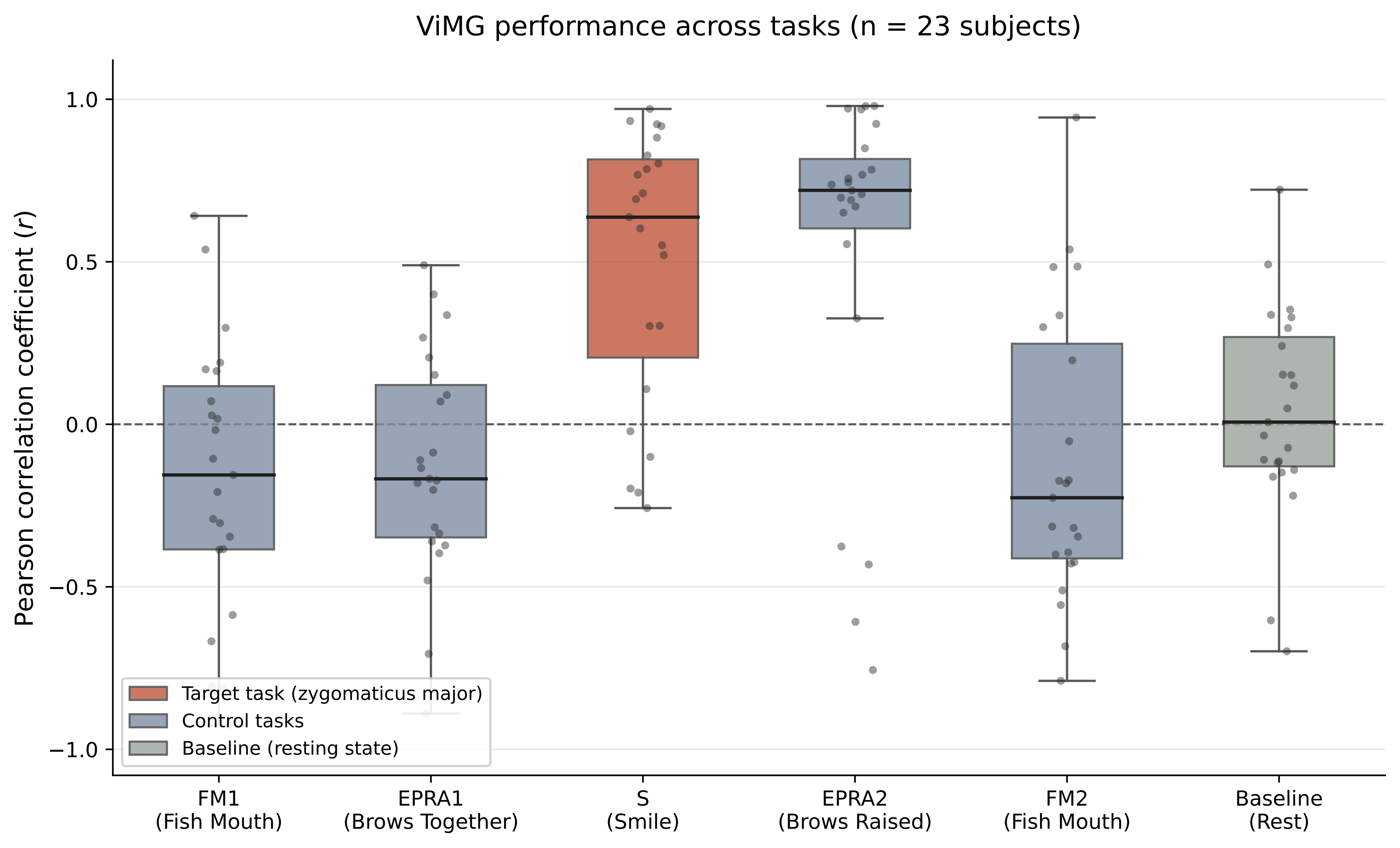

ViMG performance across tasks (n = 23 subjects)
Pearson correlation coefficient (r)
1.0
0.5
0.0
−0.5
−1.0
Target task (zygomaticus major)
Control tasks
Baseline (resting state)
FM1
(Fish Mouth)
EPRA1
(Brows Together)
S
(Smile)
EPRA2
(Brows Raised)
FM2
(Fish Mouth)
Baseline
(Rest)
