# Supplementary material for: ViMG: a contactless deep learning framework for estimating sEMG amplitude envelopes from facial videos
Source: Front Bioeng Biotechnol. 2026 Oct 30;14:1945313. doi: 10.3389/fbioe.2026.1945313 (PMC13760531; doi:10.3389/fbioe.2026.1945313)
Supplement: Supplementary file 1 [file DataSheet1.zip › results/full_model/figures/saliency/figure6_saliency.pdf]

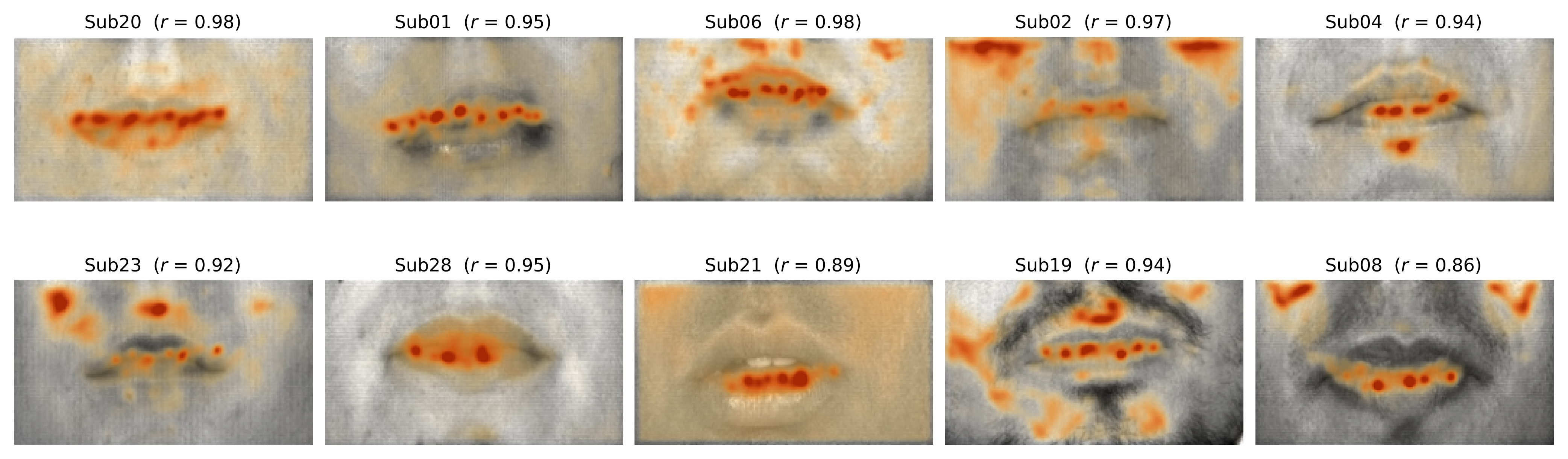
Sub20 (r = 0.98)
Sub01 (r = 0.95)
Sub06 (r = 0.98)
Sub02 (r = 0.97)
Sub04 (r = 0.94)
Sub23 (r = 0.92)
Sub28 (r = 0.95)
Sub21 (r = 0.89)
Sub19 (r = 0.94)
Sub08 (r = 0.86)
