# Supplementary material for: ViMG: a contactless deep learning framework for estimating sEMG amplitude envelopes from facial videos
Source: Front Bioeng Biotechnol. 2026 Oct 30;14:1945313. doi: 10.3389/fbioe.2026.1945313 (PMC13760531; doi:10.3389/fbioe.2026.1945313)
Supplement: Supplementary file 1 [file DataSheet1.zip › results/full_model/figures/scatter_pooled.pdf]

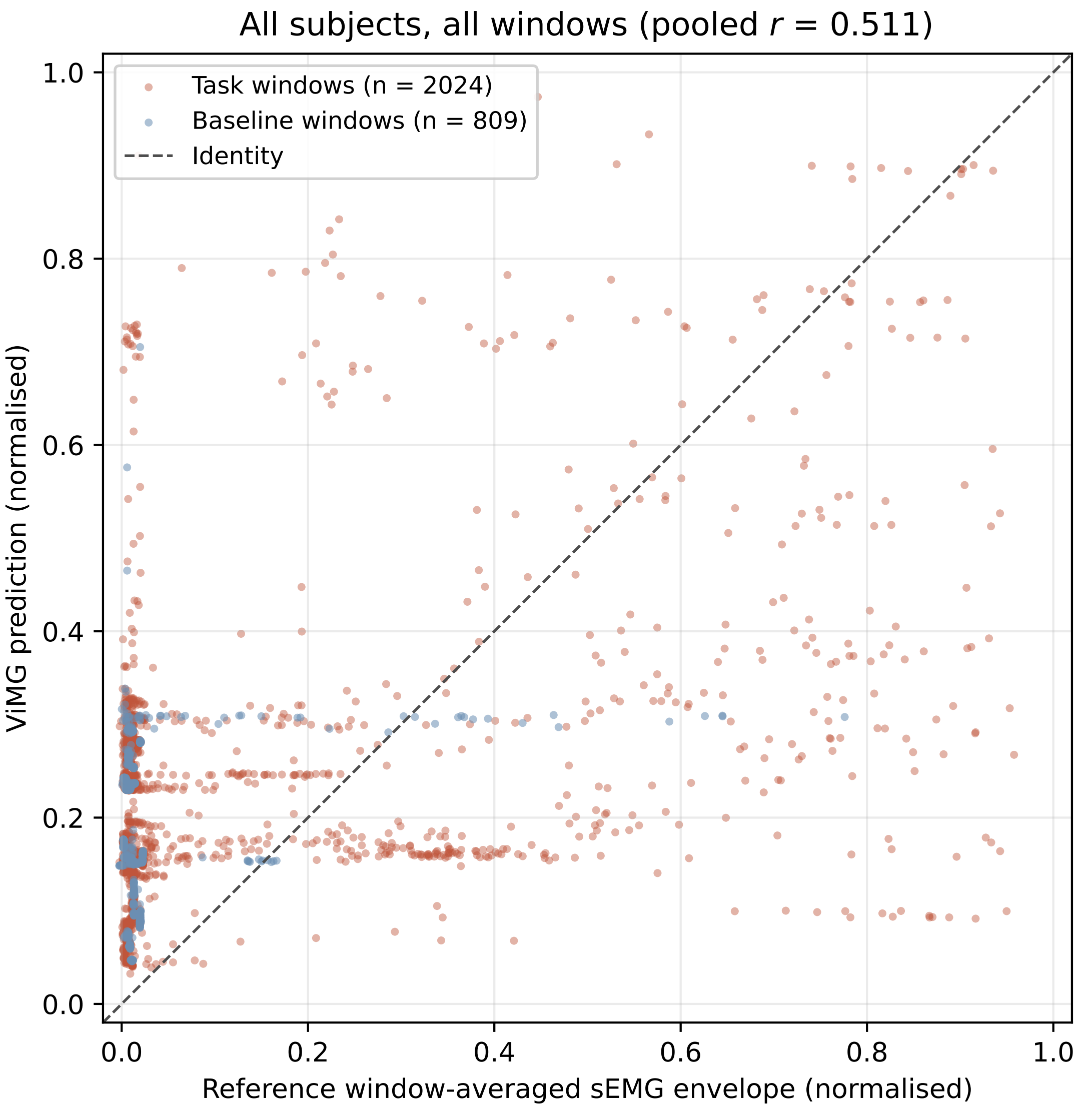

All subjects, all windows (pooled r = 0.511)
Task windows (n = 2024)
Baseline windows (n = 809)
Identity
ViMG prediction (normalised)
Reference window-averaged sEMG envelope (normalised)
0.0
0.2
0.4
0.6
0.8
1.0
