# Supplementary material for: ViMG: a contactless deep learning framework for estimating sEMG amplitude envelopes from facial videos
Source: Front Bioeng Biotechnol. 2026 Oct 30;14:1945313. doi: 10.3389/fbioe.2026.1945313 (PMC13760531; doi:10.3389/fbioe.2026.1945313)
Supplement: Supplementary file 1 [file DataSheet1.zip › results/full_model/figures/scatter_subjects.pdf]

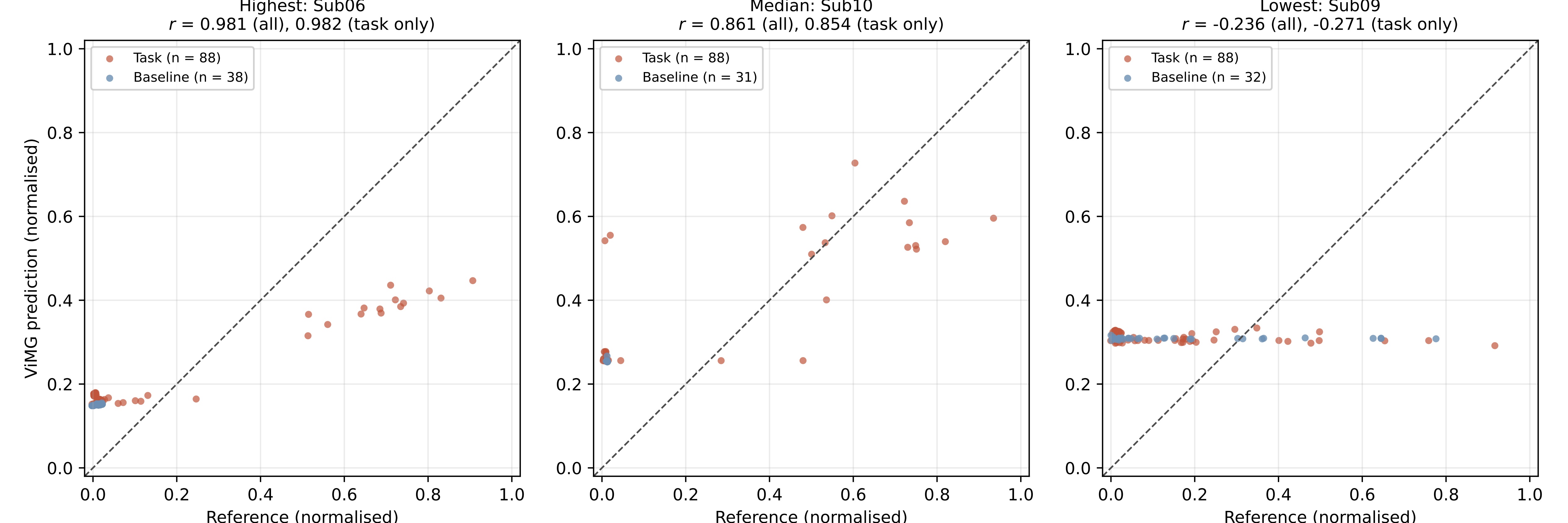
Highest: Sub06
r = 0.981 (all), 0.982 (task only)
Task (n = 88)
Baseline (n = 38)
Median: Sub10
r = 0.861 (all), 0.854 (task only)
Task (n = 88)
Baseline (n = 31)
Lowest: Sub09
r = -0.236 (all), -0.271 (task only)
Task (n = 88)
Baseline (n = 32)
ViMG prediction (normalised)
Reference (normalised)
0.0
0.2
0.4
0.6
0.8
1.0
